# Cellulose modification by recyclable swelling solvents

**DOI:** 10.1186/s13068-018-1191-z

**Published:** 2018-07-13

**Authors:** Ximing Zhang, Tianjiao Qu, Nathan S. Mosier, Lujia Han, Weihua Xiao

**Affiliations:** 1 Laboratory of Renewable Resources Engineering, Department of Agricultural and Biological Engineering, Purdue University, West Lafayette, IN 47907 USA; 2 Laboratory of Biomass and Bioprocessing Engineering, College of Engineering, China Agricultural University (East Campus), P.O. Box 191, 17 Qing-Hua-Dong-Lu, Haidian District, Beijing, 100083 People’s Republic of China

**Keywords:** Swelling agent, Trifluoroacetic acid, Phosphoric acid, Cellulose modification, Crystallinity, Enzymatic hydrolysis

## Abstract

**Background:**

The invention of efficient systems for lignocellulose conversion is essential for economically feasible production of bio-based chemicals and biofuels. One limiting step is highly selective processes to quickly decrystallize the compact cellulose structure for efficient hydrolysis. We evaluated the impact of trifluoroacetic acid (TFA) and phosphorous acid (PA)-induced swelling of crystalline cellulose on enhancement of enzymatic digestion.

**Results:**

In this study, two swelling agents, TFA and PA, are compared and found to be highly efficient for cellulose decrystallization at low temperatures within 1 h. After treatment, the microfibril structure of swollen celluloses was observed to develop distinct microscopic morphology and subsequent enzymatic hydrolysis resulted over 90% cellulose conversion within 24 h. The crystalline cellulose change was determined by reduction of loss of X-ray diffractability, and loss of resistance to enzymatic hydrolysis. NMR results suggest that both TFA and PA efficiently converted most of the crystalline cellulose regions to amorphous regions through cellulose chain relocation that inhibits recrystallization. It was found that the swelling mechanism is different between TFA and PA. To the best of our knowledge, it is the first time to compare and quantify the cellulose regions transformation by swelling agents.

**Conclusion:**

This study shows the low-temperature swelling of different celluloses in TFA and PA reduces recalcitrance of crystalline cellulose to enzymatic hydrolysis. TFA and PA are both ideal candidate swelling agents for a closed system for ease of solvent recovery by either simple distillation or filtration. This study provides potentially useful agents in large-scale deconstruction of biomass.

## Background

Cellulose is the most abundant carbohydrate polymer on earth [1]. In recent decades, utilization of cellulose to produce biofuels, and functional molecules of interests have attracted numerous attention due to its renewable, carbon neutral, and environmental friendly features [1–4]. However, cellulose, a homopolymer of glucose, has a compact fibril structure possessing high crystallinity and extensive hydrogen bonds that are formed during biosynthesis which hinders the cellulose conversion efficiency [5, 6]. Pretreatment is required to make the compact cellulose structure accessible to enzyme or chemical catalyst to efficiently hydrolyze β-1,4 glycosidic bond [7, 8]. Unlike pretreatments such as dilute acid, ammonia fiber expansion, ammonia recycle percolation pretreatment demanding high temperature or pressure, and cellulose solvent- and organic solvent-based lignocellulose fractionation (COSLIF) can fractionate lignocellulose components at modest reaction conditions [9]. The previous study shows that concentrated phosphoric acid (PA) is an effective cellulose solvent [9]. PA is a modest acid; under mild condition, cellulose can completely dissolve into it when its concentration is more than a critical value [10]. The cellulose dissolving phenomenon is known as solvent swelling effect. Similar as PA, swelling agent trifluoroacetic acid (TFA) attracts attention as it can effectively swell cellulose and be recycled due to its low boiling point, which provides an efficient and economical way to lower the cellulosic crystallinity and break up hydrogen bonds within the crystalline region [10–12].

Even at low temperatures, swelling agents can efficiently diffuse into the interstices between the fibrillary structural units of cellulose, as well as penetrate both ends into the elementary crystallites. This causes drastic changes in the crystal lattice structure [13]. As a result of swelling, cellulose crystallinity is significantly decreased, and the number and size of micro pores are increased, generating increased solvent-accessible surface area for hydrolyzing catalysts, such as enzymes [14, 15]. When swollen cellulose is further heated, gelatinization occurs which results in dissolution of the cellulose into the liquid solvent, which is a transparent liquid. Dissolution completely disrupts the cellulose crystalline supramolecular structure. Rapid precipitation of this dissolved cellulose results in a disordered, amorphous cellulose. In our previous study, TFA was used comparing the effect of adding a gelatinization and precipitation step to enhance enzymatic hydrolysis of cellulose to reducing sugars. The data reported there showed that adding gelatinization/precipitation does not further improve the cellulose reactivity from both enzymatic and chemical hydrolysis. The only significant effect was the small portion of cellulose that was hydrolyzed during gelatinization which could not be easily recovered by precipitation [15]. Our previous study determined that swelling cellulose is the key step for improving the cellulose reactivity and indicated that most of the changes relevant to enhancing hydrolysis occur through the swelling process.

To further understand the effect of swelling agents on cellulose substructure, we use two distinct celluloses in nature-cotton linters (Sigmacell, particle size ca 50 μm) and microcrystalline cellulose (Avicel PH 101, particle size ca 50 μm) as substrates. The Avicel PH 101 cellulose was prepared using sulfuric acid to hydrolyze poplar which is a representative of woody biomass and leave with more crystalline region cellulose; while the Sigmacell was prepared using herbaceous cotton linters cellulose contains both crystalline and amorphous regions cellulose. The cellulose in both untreated cotton linters and microcrystalline cellulose are in the form of cellulose I [16]. We report, for the first time, on comparisons between the physicochemical characterization of two types of cellulose by two swelling agents, TFA and PA. Both of them are known as swelling reagents for high efficiency for cellulose decrystallization which are used at high concentration and low temperature (ca 0 °C) [10, 11]. However, based on our observation, the celluloses after swelling by TFA or PA have different morphological appearances before ethanol precipitation. PA results in a transparent cellulose gel in a short time, while TFA as solvent has the unique advantage in recovery as it is readily volatile. The difference in cellulose morphology indicates that there might be differences in the swelling mechanisms. Even though both agents have been reported for cellulose conversion, no previous studies focused on study and comparison their swelling mechanism at low temperature.

## Methods

### Materials and reagents

Sigmacell cellulose (Type 50, particle size 50 µm) and Avicel^®^ PH 101 (particle size 50 µm) were purchased from Sigma-Aldrich. Trifluoroacetic acid (TFA, 99%) and phosphoric acid (PA, 86.2%) were purchased from Alfa Aesar. Ethanol (100%) was purchased from Fisher Scientific (Houston, TX).

### TFA swelling of cellulose

One gram of cellulose was mixed with 30 mL of TFA (99%) in a 50 mL plastic Falcon tube. Then, the cellulose-TFA mixture was incubated at 0 °C for a predetermined period. After swelling, approximately 60 mL of ethanol (100%) was added with vigorous stirring, resulting in washed, swollen cellulose.

The cellulose was filtered through a Whatman GF/D filter paper and washed with an additional 30 mL ethanol (80%) at a rate of 10 mL per addition to remove residual TFA. Deionized water was used to suspend the cellulose pellet which was then freeze-dried (LAGCONCO, Kansas City, MO). The TFA-swelled cellulose was collected and named as S-TFA (Sigmacell TFA-swelled) or A-TFA (Avicel-TFA-swelled) for further analysis.

### Phosphoric acid-swelled cellulose

The phosphoric acid-swelling process was based on Zhang’s [17] method with the minor modifications described below. One gram of cellulose and 3 mL distilled water were mixed in a 50 mL plastic Falcon tube, and then, 10 mL 0 °C PA (86.2%) was added to the slurry with violent agitation. The resulting mixture was kept at 0 °C for a predetermined period with stirring every 10 min. The cellulose was precipitated with ethanol, filtered, washed with additional ethanol, and then freeze-dried by the same procedure as TFA swelling. The phosphoric acid cellulose was collected and named as S-PA (Sigmacell PA swelled) or A-PA (Avicel PA swelled) for further analysis.

All six samples, untreated cellulose S-U (Sigmacell untreated) and A-U (Avicel untreated), TFA-treated cellulose (S-TFA and A-TFA), and PA-treated cellulose (S-PA and A-PA), share a similarly and extremely fine white powder appearance after freeze drying.

### Physicochemical and morphological characterization

#### Scanning electron microscopy (SEM)

Scanning electron microscopy images were obtained via Hitachi S3400N (Japan) microscope at 15 kV accelerating voltage. Prior to SEM observation, samples were placed in an ion sputter coater and subjected to gold sputtering for 0.5 h. The surface morphology of cellulose samples randomly selected and captured at 500 magnifications.

#### Atomic-force microscopy (AFM)

Protocol of AFM experiment is previously reported by Chen et al. [18].

#### X-ray diffraction (XRD)

To determine the crystallinity of cellulose before and after TFA or PA treatment, samples were analyzed by X-ray diffraction (XRD). A LabX XRD-6000 (Shimadzu) diffract meter was used to generate pattern data with Cu Kα radiation source (*λ* = 1.54060 Å) over the angular range 2*θ* = 5–40° and a step degree of 0.04. The crystalline index (CrI) was determined using the empirical method [10]:

$$ {\text{CrI}} = \frac{{I_{ 0 0 2} - I_{\text{am}} }}{{I_{ 0 0 2} }} \times 100, $$

where *I*_002_ is the maximum intensity of 002 peak at approximately 2*θ* = 22°, while *I*_am_ is the amorphous diffraction intensity at approximately 2*θ *= 18.0°.

#### Fourier transform infrared (FTIR) spectroscopy

The functional groups’ change of swelled cellulose was analyzed by FTIR. The spectra were acquired on a Perkin Elmer Spotlight 400 FTIR spectrometer (Perkin Elmer, Waltham, MA, USA) working in a transmittance mode. Before scanning, the sample pellets were prepared by mixing with spectroscopic grade KBr in a ratio of 1:100 (w/w) and then pressed under vacuum. FTIR spectra were recorded at 4 cm^−1^ resolution and 64 scans per sample in the range of 4000–500 cm^−1^.

#### Solid-state CP/MAS ^13^C NMR

Solid-state ^13^C CP/MAS NMR were performed at 100 MHz resonance frequency on a Bruker Avance DPX400 NMR spectrometer with a 4 mm CP/MAS probe and samples spinning at 5 kHz MAS speed. ^13^C CP/MAS NMR were conducted with 3 ms contact time, 30 ms acquisition time, 2 s recycle delays, and in 1600 scans. The data were analyzed by MestReNova and Origin software.

#### Cellulose reducing-end determination

The molar concentration of reducing ends of the cellulose was measured by the modified BCA method [19]. BCA reaction solution A was prepared by dissolving 0.971 g of disodium 2,2′-bicinchoninate, 27.14 g of Na_2_CO_3_, and 12.1 g of NaHCO_3_ dissolved in 500 mL of distilled water. Solution B contained 0.624 g of CuSO_4_·5H_2_O and 0.631 g of l-serine dissolved in 500 mL of water. Equal volumes of solution A and solution B were mixed to form the working solution before use. One milliliter of BCA working solution was mixed with 1 mL sample solution containing cellulose (5 mg/mL) in a tube with stopper. Glucose solutions with concentrations from 0 to 50 µM were used as standards. The mixture was incubated for 30 min at 75 °C in a convection oven. For cellulose sample, the tubes were vortex-mixed every 10 min to increase the interaction between cellulose and working solution. The tubes were rapidly cooled to 20 °C using tap water. After cooling, reaction mixtures were transferred to a centrifuge tube and centrifuged for 5 min at a speed of 10,000*g*. The supernatant was measured at 560 nm on a UV–Vis spectrometer.

### Enzymatic hydrolysis

To evaluate the enzymatic digestibility of swelled cellulose, enzymatic hydrolysis experiments were performed in 50 mL plastic round-bottom Falcon tubes in triplicate in a thermostatically controlled shaker at 50 °C and 250 rpm. The initial solid loading of 1% (w/v) was added into 5 mL, 50 mM sodium citrate buffer where pH was maintained around 4.8. Cellic™ Ctec2 (aggressive cellulase and a high level of β-glucosidase, Novozymes North America, Inc., Franklinton, NC), at an enzyme loading of 7.0 FPU/g cellulose, was used for treated and untreated samples. Sodium azide (0.2%, w/v) was added to inhibit microbial growth during hydrolysis. Samples were taken at predetermined intervals and immediately filtered through 0.2 μm nominal-pore-size nylon syringe filter (Pall, Port Washington, NY) to remove the residual substrate. Filtrate was then stored at − 20 °C until analysis by HPLC.

The glucose yield was calculated as:

$$ {\text{Glucose yield (\% )}} = \frac{{{\text{concentration of glucose }} \times {\text{ volume of reaction}}}}{\text{total mass of cellulose}} \times 100. $$

The concentration of glucose was quantified by HPLC using an HPX-87H AMINEX ion exchange column (BioRAD, Hercules, CA), a Waters 1525 pump, and a Waters 2412 Refractive Index detector (Waters, Milford, MA). The data were stored and processed using Empower™ 2 Chromatography Data Software (Waters Corp., Milford, MA). The mobile phase was 5 mM sulfuric acid in distilled, deionized water at flow rate of 0.6 mL/min. The column temperature was maintained at 60 °C. The injection volume was of 50 µL. The concentrations of glucose in the aqueous phase were determined using external calibration standards.

## Results and discussion

### Analysis of cellulose crystalline content by X-ray diffraction and FTIR spectroscopy

Crystallinity change as a function of increasing swelling time (0, 1, 12, 24, and 48 h) for both swelling agents was measured. For both PA and TFA, CrI decreased rapidly within 1 h at 0 °C and reached a stable CrI value (Table 1). For quantification purpose, 1 h was selected as swelling treatment time for fully swollen cellulose to compare the effect on cellulose substructural change. Taking into consideration of the economic feasibility and energy input for large-scale applications, temperatures lower than 0 °C were not considered as influencing factors in this study. However, temperatures of − 15 and − 20 °C (lower than the freezing point of TFA) were tested as a comparison with 0 °C in previously reported work [15]. The crystallinity indices (CrIs) reported that there were very close to our results obtained at 0 °C. Therefore, we used 0 °C as the standard swelling temperature for both TFA and PA in this work. The CrIs of the untreated Sigmacell and Avicel cellulose were 81 and 83%, respectively. TFA-treated Sigmacell and Avicel cellulose stabilized with CrI values of 42 and 38%, while PA-treated Sigmacell and Avicel were 25 and 29%, respectively. The trend of CrI changes suggests that PA disrupts cellulose crystallinity more efficiently than TFA.

**Table 1:** CrI of treated and untreated cellulose

	Sigmacell	Avicel
Untreated	81.1	83.0
TFA	42.1	37.6
PA	25.3	29.0

**Fig. 1:**
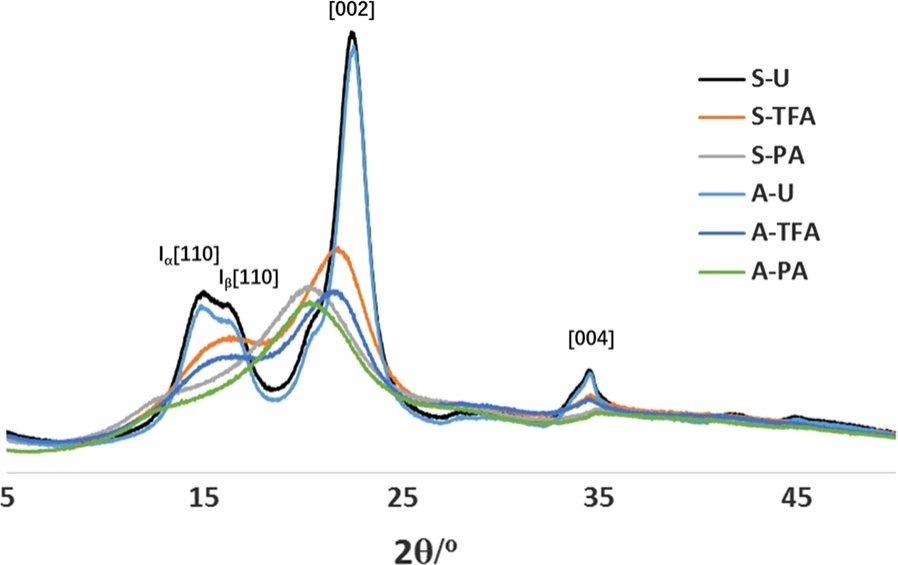
Normalized XRD diffractograms of untreated and treated cellulose

By further analyzing the XRD spectra shown in Fig. 1, it further confirms that loss of crystallinity during swelling results in cellulose crystal transmutation. The untreated celluloses were in cellulose I polymorph patterns. From the spectra, both cellulose *I*_*α*_ and cellulose *I*_*β*_ coexist in the swelled samples. As shown with the designated peaks, 2*θ* = 16° represents the cellulose *I*_*α*_ [1 1 0] lattice plane, and 18° represent cellulose *I*_*β*_ [1 1 0] lattice plane, 22.7°, and finally, 35° corresponds to the [0 0 2] and [0 0 4] lattice planes. After the swelling process, TFA- and PA-treated celluloses had distinct spectra shifts, indicating different forms of cellulose crystal transformations.

For the TFA-treated celluloses, the cellulose *I*_*α*_ [1 1 0] lattice plane was shifted from 2*θ* value of 16° to a higher 2*θ* value, merging with cellulose *I*_*β*_ 2*θ* value. The intensity for [0 0 2] lattice plane was significantly decreased and shifted angle to a lower 2*θ* value. For the PA-treated celluloses, the intensity for [0 0 2] lattice plane was further weakened compared to TFA-treated celluloses and shifted to a lower 2*θ* value. It is noticed that cellulose *I*_*α*_ and *I*_*β*_ [1 1 0] plane lattice signals were not detected, while [1 1 0] lattice plane with 2*θ* value 12.2° was observed for both A-PA and S-PA. TFA-treated celluloses did not have 2*θ* values for [1 1 0] that corresponded to PA-treated celluloses. Both PA- and TFA-treated spectra show the transition of cellulose I to cellulose II. PA treatment resulted in a 2*θ* value of 12°, representing the [1 1 0] lattice plane of cellulose II. TFA treatment resulted in a cellulose I [1 1 0] increased angle shift from 16.5° to close to [2 0 0] [20–23].

These data show that the cellulose I structure was disrupted and resulted in more disorder cellulose structure [24]. According to our previous study, cellulose II was formed by recrystallization of swollen cellulose when freeze-dried [15]. However, distinct spectra patterns between TFA- and PA-treated cellulose suggest that, besides recrystallization, there may be different effects on the crystal structure between the two swelling agents.

**Fig. 2:**
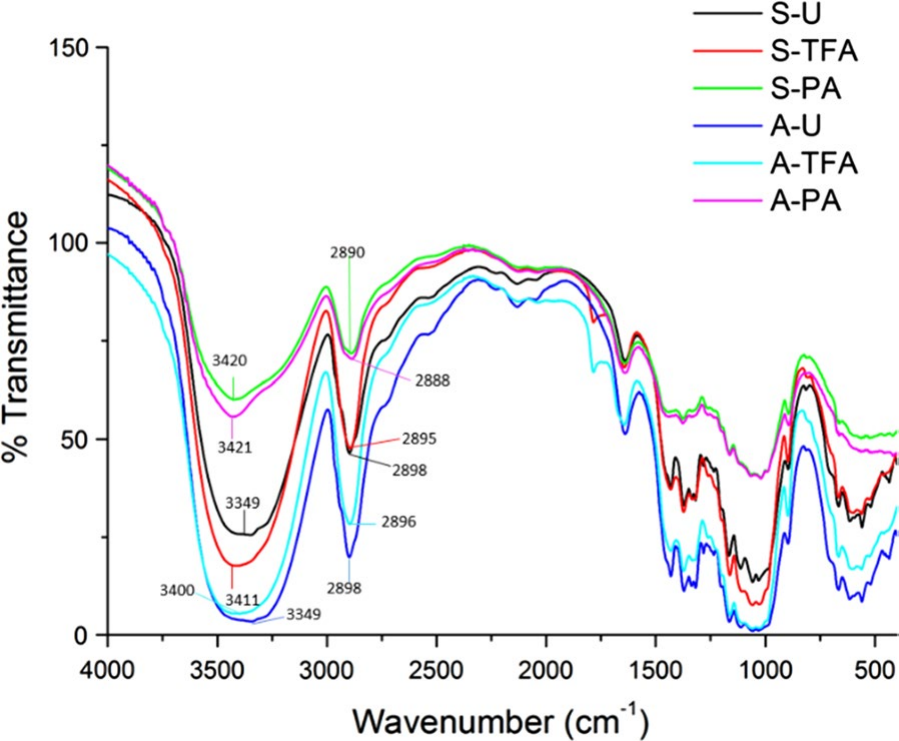
FTIR spectroscopy analysis of the cellulose substrates

To further examine the changes to the cellulose structure, FTIR analyses of the samples were performed (Fig. 2 and Table 2). Related to the crystallinity change before and after the swelling process, O–H stretching and C–H stretching were the two most significant signals that can be observed in the FTIR spectra.

**Table 2:** FTIR peaks’ wavenumber and absorbance

S-U	3349.60 cm^−1^/0.59 A	2898.34 cm^−1^/0.33 A
S-TFA	3411.22 cm^−1^/0.75 A	2895.08 cm^−1^/0.32 A
S-PA	3420.61 cm^−1^/0.22 A	2890.04 cm^−1^/0.14 A
A-U	3349.19 cm^−1^/1.46 A	2898.81 cm^−1^/0.70 A
A-TFA	3400.10 cm^−1^/1.26 A	2896.17 cm^−1^/0.54 A
A-PA	3421.90 cm^−1^/0.25 A	2888.99 cm^−1^/0.15 A

The O–H stretching is observed at the absorbance of 3600–3100 cm^−1^ which includes valence vibration of hydrogen-bonded OH groups, i.e., the intramolecular hydrogen bond, intermolecular hydrogen bond, and the O–H stretching. The signal intensities are significantly weakened by PA swelling, while TFA swelling does not result in as significant change as PA does. The O–H stretching corresponds both to the larger CrI decrease by PA than by TFA and to the XRD spectra results. Both TFA and PA can significantly decrease the cellulose structure crystallinity, while decrystallization by PA was more complete than by TFA.

The C–H stretching signal indicates a high percentage of long-chain hydrocarbons in the sample. Decreasing signal for C–H stretching (2895 cm^−1^) was observed after the swelling process in both substrates. PA decreased more than TFA. This is consistent with what was observed in the XRD results.

The drastically decrease of crystallinity of cellulose and lignocellulosic substrates to more amorphous cellulose regions was also observed by treating cellulose residue using ionic liquids, confirming that both swelling agents and ionic liquids could enhance the cellulose conversion efficiency by breaking the cellulose microstructure hydrogen bond to increase the accessible area [24].

### Study redistribution and recrystallization of cellulose content by CP/MAS ^13^C NMR

In addition to XRD and FTIR, CP/MAS ^13^C NMR provided insight into cellulosic substructural changes from swelling treatment. Larsson et al. [25] and Wickholm et al. [26] determined spectral fitting methods and assignment for cellulose using cotton linter and several other cellulose sources. They found that the deconvoluted NMR signal of C-4 region (80–92 ppm) gives key information on the specific cellulose fibril structure. The C-4 region divided into two distinct region ranges: crystalline region and amorphous region [27]. Region from 86 to 92 ppm represents cellulose in more ordered forms. It is crystalline and defined separately as *I*_*α*_ at 89.35 ppm, *I*_(*α*+*β*)_ at 88.5 ppm, and *I*_*β*_ at 87.91 ppm. A fourth form of cellulose with more mobility was determined and named as paracrystalline at 88.25 ppm. It was in-core or beneath fibril with less-ordered crystalline form compared to the others. All of the four crystalline regions are surface inaccessible to the surrounding solvent.

Region from 80 to 86 ppm represents more disordered forms or amorphous cellulose. They are defined as (solvent) accessible fibril surface, (solvent) inaccessible fibril surface and cellulose oligomers, respectively [26]. Solvent-accessible fibril surface at 84.95 and 83.26 ppm is determined to be amorphous cellulose at fibril or crystallite surfaces accessible to surrounding solvent [28, 29]. In contrast, inaccessible fibril surface at 83.43 ppm are defined as amorphous form cellulose portion which is inaccessible to the surrounding solvent. Cellulose oligomers at 81.68 ppm are defined as less-ordered forms of carbohydrates or hemicellulose residues [25].

**Fig. 3:**
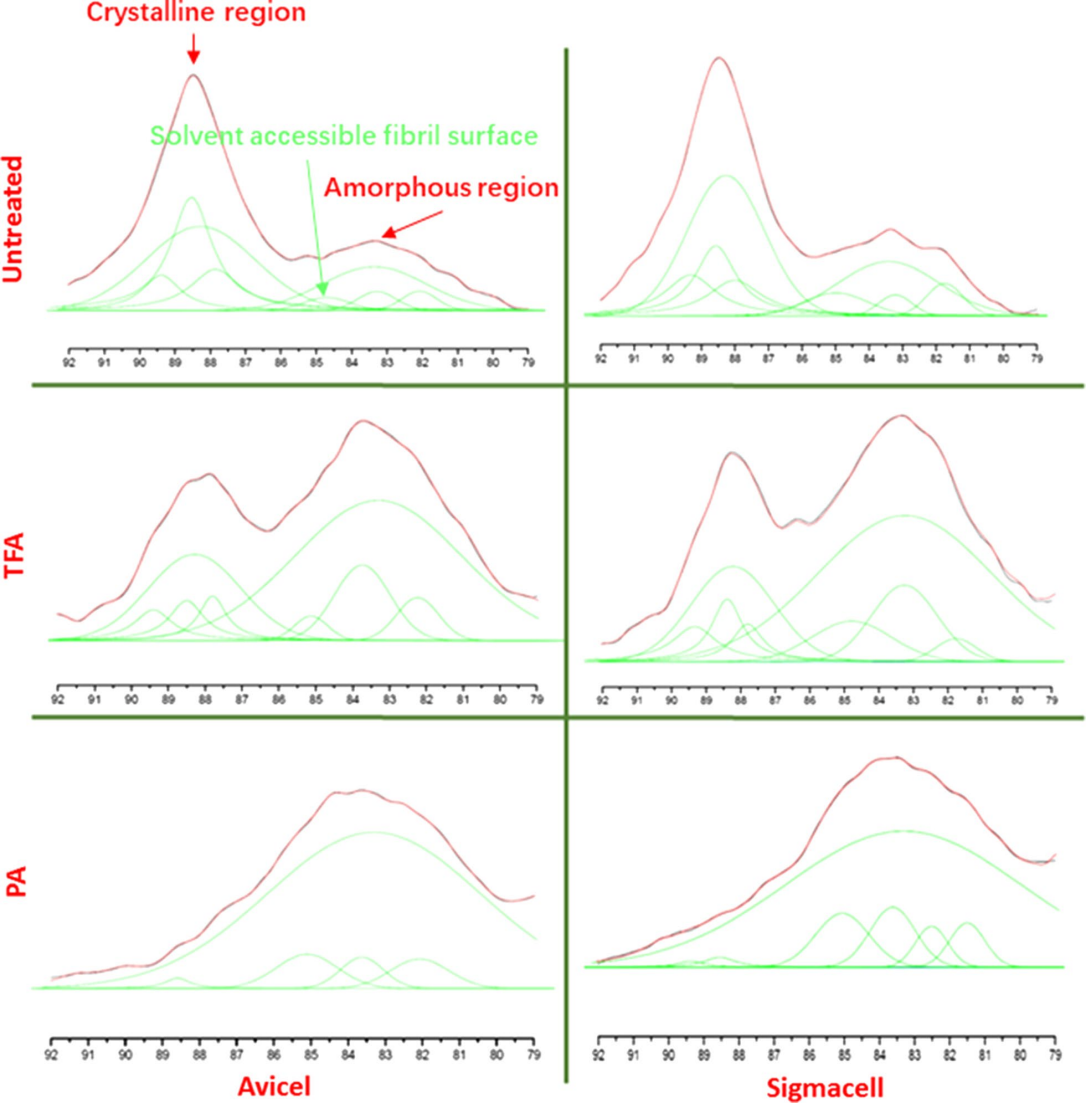
NMR C-4 region spectra of treated and untreated cellulose. Spectra fitting was used to analysis. The red line represents the experimental spectrum. The fitted spectral lines are shown as green. The results of the assignments are given in Table 3

From Fig. 3 and Table 3, the NMR spectra showed that untreated celluloses had a dominant crystalline peak compared to the amorphous region. After TFA swelling process, the amorphous region was significantly enhanced, while the crystalline region decreased. The peaks change trends correspond to the CrI decrease after TFA swelling treatment. After PA treatment, more severe decrease of the crystalline region could be observed. The dominant peak fell on amorphous region. With the transition of the crystalline peaks to the amorphous peaks, the CrI value of both PA-treated sample decreased more substantial than the TFA-treated sample (Table 1). The CrI value changes indicate the H-bonding rearrangement undergoing within the cellulose structure for both TFA-treated and PA-treated sample. The PA treatment has more thoroughly change in the cellulose substructure than TFA swelling treatment, which can be further confirmed and analyzed by the deconvoluted peaks.

**Table 3:** Assignments of signals in the C-4 region of the untreated and treated cellulose spectra

	Chemical shift (ppm)	Line shape
*I*_*α*_	89.35 (0.03)^a^	Lorentz
*I*_( *α*+ *β*)_	88.50 (0.10)	Lorentz
Paracrystalline	88.25 (0.03)	Gauss
*I*_*β*_	87.91 (0.15)	Lorentz
Accessible fibril surfaces	84.95 (0.14)	Gauss
Inaccessible fibril surfaces	83.43 (0.18)	Gauss
Accessible fibril surfaces	83.26 (0.06)	Gauss
Cellulose oligomers	81.68 (0.15)	Gauss

^a^Values in brackets are standard errors

**Fig. 4:**
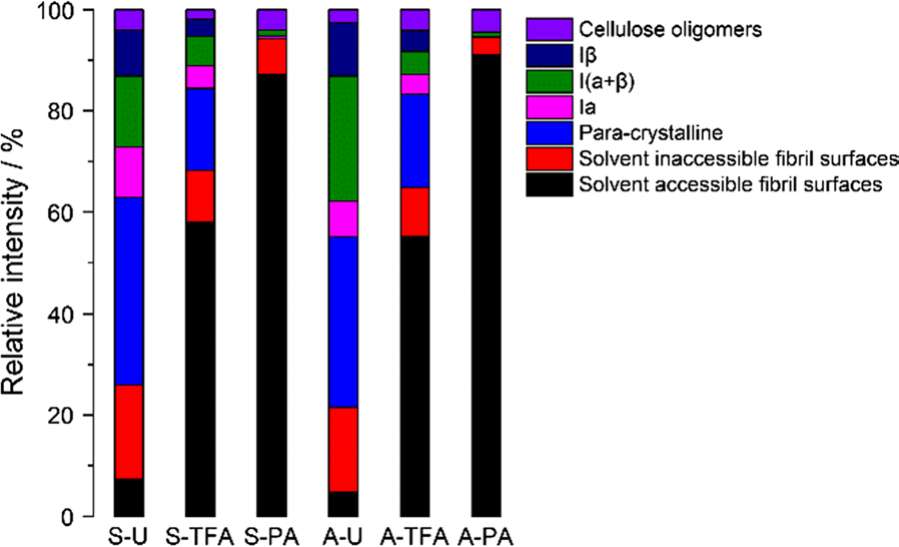
Relative signal intensities obtained by fitting of C-4 region spectra of untreated and treated cellulose

Deconvoluted peaks (green line) show the details of assignment and spectra fitting of CP-MAS NMR signal in the C-4 region before and after swelling (Table 3 and Fig. 3). As the crystalline and amorphous region signals are complementary, the deconvoluted peak signals are integrated with total intensity of 100%. Figure 4 compares relative signal intensities using the deconvoluted peak results of Fig. 3. Untreated cotton linter has a large percentage which includes crystalline and paracrystalline portion. For the amorphous portion, the inaccessible fibril surfaces are larger than accessible fibril surfaces. After TFA treatment, the accessible fibril surface was dramatically increased by 50.6% with the inaccessible fibril surface portion and crystalline and paracrystalline regions decreased, indicating the disruption of rigidly crystal cellulose construction into amorphous one. PA treatment further increased the accessible region while maintaining similar inaccessible fibril surface fraction. However, accessible fibril surfaces increase to 87.2%. Most of the crystalline and paracrystalline regions declined to approximately 2% in total. It is worth noting that the relative intensities among crystalline region *I*_*α*_, *I*_(*α*+*β*)_, and *I*_*β*_ was not significantly changed. This is likely due to the low temperature of the swelling treatment, as solid-state conversion of *I*_*α*_ to *I*_*β*_ requires high temperature for transforming the crystals [30]. Interestingly, when Avicel is used as cellulose substrate, a similar transition is observed.

Overall, the inner structure of cellulose was disrupted after being treated by TFA, whereas PA treatment can further improve accessible fibril surfaces region into dominant percentage.

### Analysis of crystalline cellulose change by microscopy

**Fig. 5:**
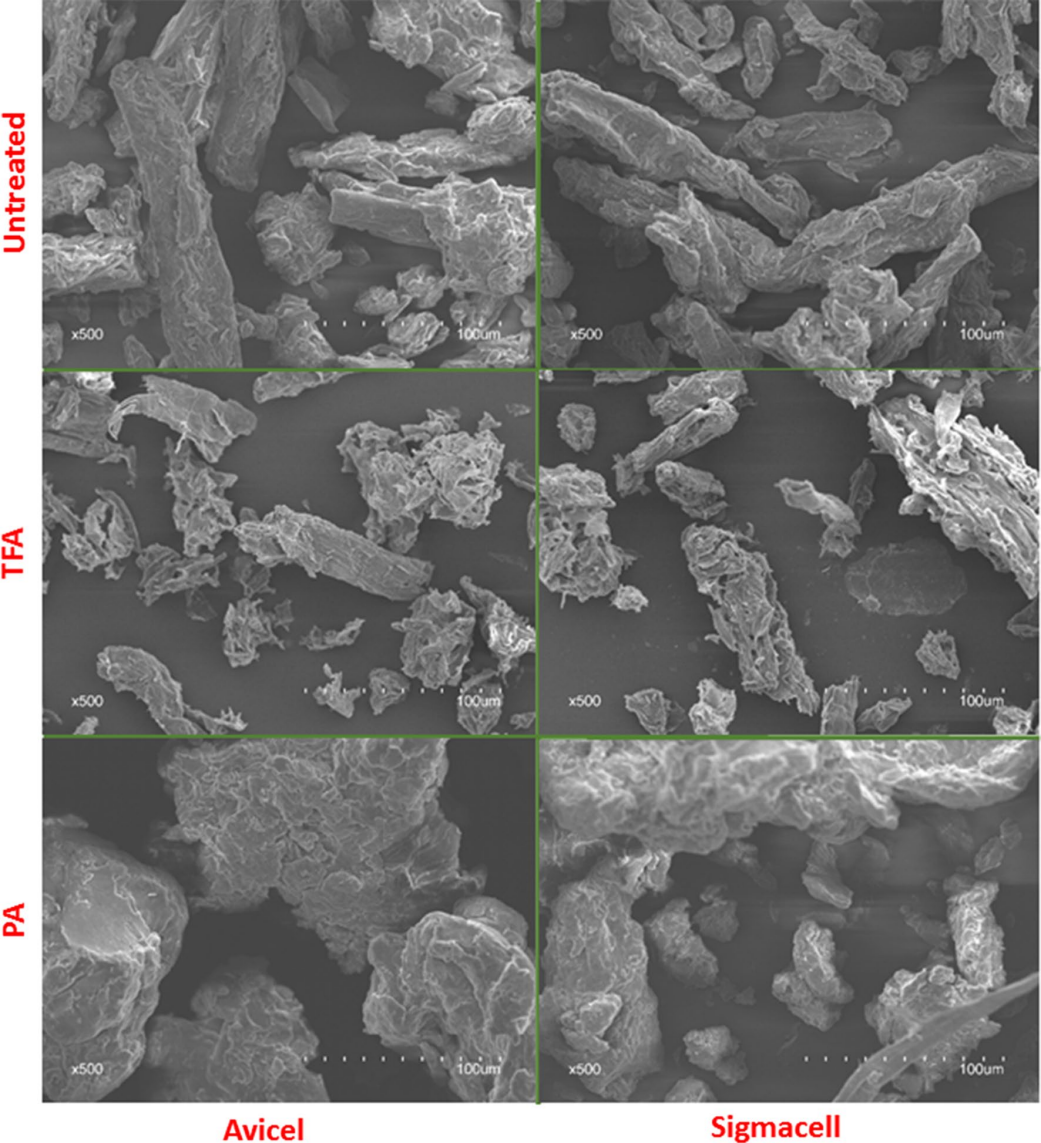
Scanning electron microscopy (SEM) images of native and swollen substrates

Scanning electron microscopy was used to evaluate changes in surface morphology of cellulose substrate before and after swelling. Figure 5 shows the SEM images at 500× magnification. Untreated cellulose particles are generally rod-shaped and with orderly arranged fibers observable on the cellulose surface.

After swelling with TFA, a significant morphology change was observed compared to untreated cellulose substrates. The surface of the particles was more irregular with some fibers visible protruding from the surface. This confirms the previous results which showed that the TFA dimer at low temperature can penetrate the cellulose structure to swell the cellulose [11].

The phosphoric acid treatment did not generate the same morphological surface change as TFA treatment. The particles were distributed loosely after PA swelling, while the surface of the particles was similar to the untreated cellulose. This is in contrast to the XRD and FTIR data which show greater changes during PA treatment.

**Fig. 6:**
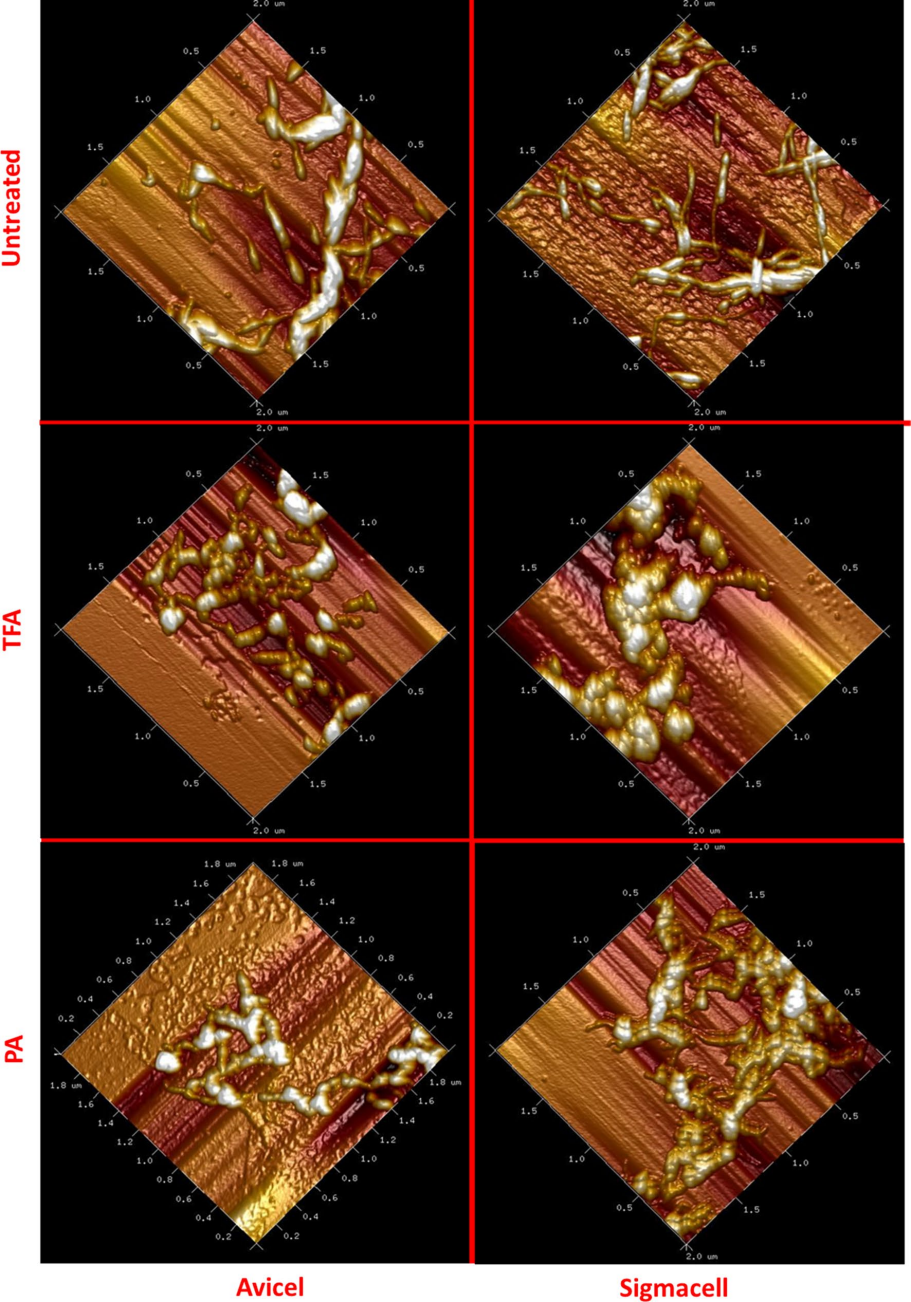
AFM analysis of native and swollen substrates

Different swelling mechanisms between TFA and PA were also observed by atomic-force microscopy (AMF). Figure 6 shows the AMF images of the native and swollen cellulose substrates. Untreated cellulose has longitudinal and rod-shaped particles. Untreated microcrystalline cellulose (Avicel) has a larger diameter compared to cotton linters. After the TFA treatment, the length of microcrystalline cellulose was dramatically reduced to shorter fractions and loosely distributed with a more spherical shape due to TFA swelling. Sigmacell cotton linters with native amorphous regions provide unique advantage for the TFA molecules penetration and generate large sphere interconnected swollen cellulose. The length of the TFA-treated cotton linters does not change significantly.

The PA-swollen Avicel and Sigmacell celluloses were observed with cellulose redistribution and recrystallization with thinner-shaped rod. Celluloses are more tightly packed compared to loosely distributed TFA-treated celluloses. The AFM images also show that swollen effect of PA is not as obvious as TFA, in contrast to the XRD and FTIR data which show the opposite.

### Rates of enzymatic hydrolysis of swollen cellulose

**Fig. 7:**
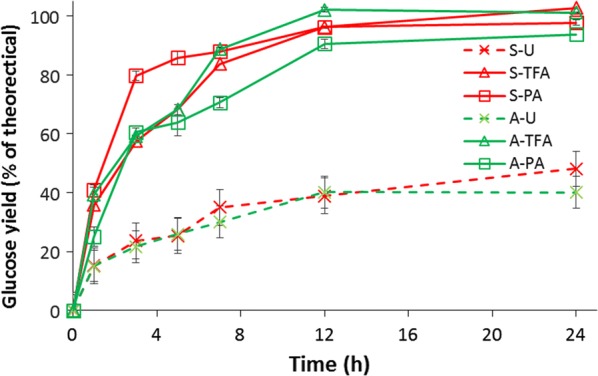
Rates of enzymatic hydrolysis of untreated and swelled cellulose with Cellic^®^ Ctec2 (Novozymes) loading 7 FPU/g glucan

The analyses described above show significant decreases in cellulose crystallinity and increases in solvent-accessible cellulose surface, both of which correlate strongly with cellulose digestibility by enzymes [31, 32]. We have confirmed this by performing enzymatic hydrolysis of the untreated, TFA-treated, and PA-treated cellulose samples (Fig. 7). Prior to swelling, the saccharification rates and yields of both varieties of cellulose are low (ca 40%) through 24 h of reaction. The results are consistent with the previous reports and indicate that cellulose is partially accessible due to their compact crystalline structure [10]. The untreated Sigmacell yields 6% more glucose compared to Avicel, which might be due to the amorphous regions of cotton linters compared to high crystallinity of Avicel.

After treatment by PA or TFA, the enzymatic hydrolysis yields were dramatically improved for both varieties of cellulose, likely due to the reduced crystallinity. PA-treated cellulose has a higher initial kinetic rate (0–3 h) compared to TFA-treated cellulose. This corresponds to the higher percentage of solvent-accessible fibril surfaces in PA-treated celluloses compared to TFA-treated celluloses. Interestingly, PA-swelled Sigmacell has a higher glucose yield compared to PA-swelled Avicel, while maintaining a similar initial hydrolysis rate. This can be interpreted that, even after swelling, the microcrystalline Avicel that is manufactured by sulfuric acid hydrolysis of wood pulp retains some of its recalcitrance after PA swelling.

Compared to PA-treated cellulose, TFA-treated Avicel and Sigmacell do not have a significant difference both on trend of glucose yield and reaction rates. Both of the TFA-treated celluloses were fully converted into glucose by 24 h. However, the slower rates during the initial hydrolysis for TFA-treated cellulose correlated to the lower fraction of fibril that was solvent accessible compared to PA-treated celluloses. As the surface of the TFA-treated cellulose was hydrolyzed, the newly exposed paracrystalline and cellulose oligomers were quickly digested.

### Analyses of swelling agent effect on celluloses

Low-temperature swelling by TFA and PA results in efficient disruption of cellulose crystallinity. TFA is recommended as it has a wide operational condition (− 20 to 78 °C) and distillation will be feasible after the swelling process. Compared to TFA, PA is unique that during swelling process, cellulose becomes a gel in a short time. PA may be also recyclable after the swelling process.

X-ray diffraction results showed that, after swelling, CrI indices were decreased significantly for both swelling agents. PA has a larger decrease in CrI. More importantly, XRD spectra shifts showed that PA and TFA may have different swelling mechanisms. The SEM and AFM results showed different morphological changes after the TFA (flaky on the surface of the swelled cellulose), while PA treatment does not generate as significant change as the surface of TFA-swelled cellulose. Furthermore, AFM showed that TFA treatment results in predominantly spherical particles, while PA generated cylindrical morphology. The FTIR results confirmed that the intramolecular bonding was changed during treatment. The different effects and extent of cellulose microstructure changes are determined and analyzed by deconvolution of NMR data. The results clearly showed that choice of swelling agent is more important than cellulose source for determining the type and extent of changes to the cellulose.

Enzymatic hydrolysis is the most effective way to evaluate the resulting change in cellulose reactivity after swelling. We found that the early stage (< 6 h) hydrolysis of phosphoric acid-swelled cellulose has significantly higher initial hydrolysis rates compared to TFA. Regardless of cellulose type, phosphoric acid can convert more of the cellulose into accessible fibril surface, with 29% higher accessible surface than TFA. This might explain why the phosphoric acid treated cellulose has faster initial hydrolysis rates than TFA-treated cellulose. Combined with the results of sugar reducing ends after swelling process, as shown in Table 4, TFA has a more significant effect on exposing increased sugar reducing ends at low temperature, while PA mainly physically relocates the cellulose from largely solvent inaccessible to more solvent accessible. This might be explained by the fact that concentrated PA has a freezing point slightly below 4 °C. TFA has less effect on changing cellulose crystal forms, but has a higher impact on decreasing the degree of polymerization of cellulose.

**Table 4:** Reducing ends of the untreated and treated cellulose

Sample	Reducing sugar concentration (μM)	Standard deviation
A-U	11.2	0.3
A-TFA	47.6	2.1
A-PA	9.8	1.2
S-U	12.3	0.9
S-TFA	55.3	5.3
S-PA	10.2	0.8

Taken together, these data give us insights into different mechanisms for TFA and phosphoric acid-swelling process. CrI change of cellulose is not the only indicator on cellulose reactivity, and more importantly, cellulose substructure change should be taken into consideration, since swelling agents function differently.

Both TFA and PA treatments resulted in a significant cellulose decrystallization. We used CP/MAS ^13^C NMR as a non-destructive tool to further quantify changes to the cellulose super-molecular structure upon TFA and PA treatment [26]. We found the spectral fitting of NMR spectrum of cellulose I signal cluster between 80 and 92 ppm which have significant different strength between the TFA- and PA-treated cotton linters and microcrystalline cellulose [26]. The NMR results confirmed higher solvent-accessible fiber region of PA treatment cellulose correlate to higher initial hydrolysis rates for cellulose by enzymes. TFA caused significant changes to the microscopic morphology of the cellulose character compared to PA-treated cellulose, TFA-treated cellulose presented a lower percent of the total portion that was solvent accessible but has a much higher amount of reducing sugar ends, suggesting partial hydrolysis of the cellulose during treatment.

To sum up, swelling mechanisms between PA and TFA lie in three ways. First, morphologically, PA generates spherical swollen cellulose, TFA generates cylindric swollen cellulose. The morphology of the swollen celluloses indicates that PA results in a more thoroughly swelling change in cellulosic substructure as more space was created in the sample (confirmed by XRD results). Second, chemically, the PA is more efficient in creating inner space by breaking inter cellulose chain hydrogen bonds than hydrolyzing 1,4-glycosidic bonds in cellulose chain, while TFA functions as dimer to create more porous cellulosic structure under low temperature, as well as generated much higher sugar reducing ends content than PA. Third, effects on enzymatic hydrolysis conversion efficiency of two swelling agents demonstrated that creating cellulose inner space between cellulose chain is more important than creating sugar reducing ends for anchoring biocatalyst active site.

## Conclusions

The results demonstrated that TFA and PA have different mechanisms on cellulose modification, but result in similar yields during enzymatic hydrolysis after 24 h. Hydrolysis data showed that cellulose after TFA treatment has more homogeneity as TFA catalyzed and exposed cellulose with sugar reducing ends. PA treatment has more impact on cellulose internal fibril structure at low temperature, but does result in more cellulose chain ends as determined by exposed reducing sugars. The swelling process demonstrated that crystallinity change is not the only indicator for cellulose modification and reactivity improvement, while cellulose substructure change is more important.
